# Longitudinal Assessment of Tau Pathology in Patients with Alzheimer’s Disease Using [^18^F]THK-5117 Positron Emission Tomography

**DOI:** 10.1371/journal.pone.0140311

**Published:** 2015-10-13

**Authors:** Aiko Ishiki, Nobuyuki Okamura, Katsutoshi Furukawa, Shozo Furumoto, Ryuichi Harada, Naoki Tomita, Kotaro Hiraoka, Shoichi Watanuki, Yoichi Ishikawa, Tetsuro Tago, Yoshihito Funaki, Ren Iwata, Manabu Tashiro, Kazuhiko Yanai, Yukitsuka Kudo, Hiroyuki Arai

**Affiliations:** 1 Department of Geriatrics and Gerontology, Institute of Development, Aging and Cancer, Tohoku University, Sendai city, Miyagi prefecture, Japan; 2 Department of Pharmacology, Tohoku University School of Medicine, Sendai city, Miyagi prefecture, Japan; 3 Division of Neuro-imaging, Institute of Development, Aging and Cancer, Tohoku University, Sendai city, Miyagi prefecture, Japan; 4 Division of Radiopharmaceutical Chemistry, Cyclotron and Radioisotope Center, Tohoku University, Sendai city, Miyagi prefecture, Japan; 5 Division of Cyclotron Nuclear Medicine, Cyclotron and Radioisotope Center, Tohoku University, Sendai city, Miyagi prefecture, Japan; Nathan Kline Institute and New York University School of Medicine, UNITED STATES

## Abstract

The formation of neurofibrillary tangles is believed to contribute to the neurodegeneration observed in Alzheimer’s disease (AD). Postmortem studies have shown strong associations between the neurofibrillary pathology and both neuronal loss and the severity of cognitive impairment. However, the temporal changes in the neurofibrillary pathology and its association with the progression of the disease are not well understood. Tau positron emission tomography (PET) imaging is expected to be useful for the longitudinal assessment of neurofibrillary pathology in the living brain. Here, we performed a longitudinal PET study using the tau-selective PET tracer [^18^F]THK-5117 in patients with AD and in healthy control subjects. Annual changes in [^18^F]THK-5117 binding were significantly elevated in the middle and inferior temporal gyri and in the fusiform gyrus of patients with AD. Compared to patients with mild AD, patients with moderate AD showed greater changes in the tau load that were more widely distributed across the cortical regions. Furthermore, a significant correlation was observed between the annual changes in cognitive decline and regional [^18^F]THK-5117 binding. These results suggest that the cognitive decline observed in patients with AD is attributable to the progression of neurofibrillary pathology. Longitudinal assessment of tau pathology will contribute to the assessment of disease progression and treatment efficacy.

## Introduction

The increasing age of the population is leading to an increase in the prevalence of dementia. According to the 2009 World Alzheimer report, an estimated 35.6 million people were living with dementia at a total cost of more than US$600 billion in 2009. Alzheimer’s disease (AD), the most common cause of dementia, is neuropathologically defined by two characteristic protein deposits in the brain: senile plaques and neurofibrillary tangles (NFTs) [1]. Senile plaques are composed of extracellular aggregates of amyloid-β (Aβ) protein, while NFTs are composed of twisted filaments, termed paired helical filaments, of hyperphosphorylated tau protein [2]. Post-mortem studies have reported that NFT lesions initially appear in the transentorhinal cortex, and then extend to the hippocampus, temporal cortex, and other neocortical areas over time [1]. According to the amyloid cascade hypothesis [3], the accumulation of Aβ is a primary feature of AD, which is followed by the accumulation of NFTs and eventually neuronal death. Thus, both Aβ and tau are excellent targets for developing treatments for AD.

Many candidates for anti-amyloid and anti-tau drugs have been developed to reduce the amount of Aβ and tau in the brain [4,5]. However, to promote the development of innovative drugs that target the Aβ and tau proteins, we first need to establish a method of evaluating the amounts of these proteins in the brain to serve as outcome measures in clinical trials of disease-modifying agents. Information on the Aβ and tau loads will also help to predict future cognitive decline in non-demented individuals. Preclinical amyloid pathology has been investigated using cerebrospinal fluid and amyloid positron emission tomography (PET) [6,7]. As for the tau protein, tau and phosphorylated-tau levels in the cerebrospinal fluid have been used as biomarkers of neurodegeneration in patients with AD [8,9]. Recently, several compounds that can act as tau-selective PET tracers have become available and are being tested in living subjects [10–16]. Post-mortem studies have shown that NFTs are highly associated with neuronal loss and the severity of cognitive decline [17,18], while senile plaques appear at the presymptomatic stage and their levels stabilize relatively early in the disease process [19]. Tau PET imaging is expected to help measure the pathologic time course of NFT formation in the human brain, and thus may aid in disease staging, drug development, and in measuring treatment outcomes as a surrogate marker [20].

Previously, we developed ^18^F-labeled arylquinoline derivatives, including [^18^F]THK-5117, as tau-selective PET tracers and succeeded in visualizing NFT pathology [10,12,15]. Although these cross-sectional studies elucidated the differences in the spatial distribution of NFTs between patients with AD and healthy controls (HCs), no reports exist on the longitudinal changes in the spatial distribution of tau deposition in the human brain. Here, we performed a longitudinal PET study using a novel tau-selective PET tracer, [^18^F]THK-5117, in order to investigate the sequential changes in the spatial distribution of tau and its association with cognitive decline in patients with AD.

## Materials and Methods

### Participants

A total of 10 participants, including five patients with AD and five age-matched HCs, participated in the longitudinal studies. The studies consisted of serial cognitive assessments, MR imaging (MRI), and [^18^F]THK-5117 PET scans. Diagnosis of probable AD was based on the criteria outlined by the National Institute of Neurological and Communicative Disorders and Stroke and the Alzheimer’s disease Related Disorders Association. All of the patients with AD had amyloid deposition on their PiB-PET scans at baseline. The HCs were asymptomatic, cognitively normal volunteers who did not have any observable cerebrovascular lesions on their MRI scans or any amyloid deposition on their PiB-PET scans at baseline. All participants were screened for their medical history and cognitive performance by neurologists and a neuropsychiatrist. The MMSE and ADAS-cog were used to assess the global cognitive performance of the participants. Participants were followed for 1.3 ± 0.2 years (range: 1.2–1.5 years) and then re-examined. These examinations were performed under the regulations of the Ethics Committee of Tohoku University Hospital (approval number: 2013-2-52). After providing the participants with a complete description of the study, written informed consent was obtained from each participant or their guardians.

### Radiosynthesis for the clinical PET study

[^18^F]THK-5117 and [^11^C]PiB were prepared in the Cyclotron and Radioisotope Center at Tohoku University. [^18^F]THK-5117 was synthesized by nucleophilic substitution of the tosylate precursor, (2-(4-methylaminophenyl)-6-[[2-(tetrahydro-2H-pyran-2-yloxy)-3-tosyloxy]propoxy] quinoline, as described previously. Injectable solutions of [^18^F]THK-5117 were prepared with a radiochemical purity of >95% and a specific activity of 357 ± 270 GBq/μmol. [^11^C]PiB was synthesized through a loop method using ^11^C-methyl triflate, as reported previously[15].

### PET and MRI

PET imaging was performed using an Eminence STARGATE scanner (Shimadzu, Kyoto, Japan). After injecting 185 MBq of [^18^F]THK-5117 or 296 MBq of [^11^C]PiB, dynamic PET images were obtained for 90 min or 70 min, respectively. MR scans were performed in all subjects. T1-weighted and T2-weighted MR images were obtained using a SIGNA 1.5-Tesla machine (General Electric, Milwaukee, WI). [^18^F]THK-5117 PET images from 60 to 80 min post-injection were used for the following analysis.

### Image analysis

The PNEURO module in the PMOD software (version 3.6; PMOD Technologies, Zurich, Switzerland) was used to place and evaluate the volumes of interest (VOIs). The T1-weighted MR images acquired from each subject were initially segmented into gray matter, white matter, and cerebrospinal fluid; gray matter probability maps were calculated. The PET images were matched rigidly to the MR images. The MR images were spatially normalized to the Montreal Neurological Institute T1 template. VOIs were automatically outlined on the normalized MR images according to the Hammers maximum probability atlas, which is implemented in the PMOD software. VOIs were defined in the following regions: hippocampus, parahippocampal gyrus, fusiform gyrus, middle and inferior temporal gyri, superior parietal cortex, lateral occipital cortex, and cerebellar cortex. The cerebellar gray matter was used as a reference region. The ratio of the regional SUV to the cerebellar cortex SUV (SUVR) was used as an index of tracer retention.

### Statistical analysis

Mann-Whitney U tests were applied to identify group differences in the clinical variables. Group differences in annual rate of change in regional [^18^F]THK-5117 retention ratio were evaluated by analysis of variance (ANOVA) followed by the Bonferroni’s multiple comparison test. Spearman’s rank correlation coefficients were calculated to access the relationship between the annual changes in the ADAS-cog scores and the [^18^F]THK-5117 SUVR. The results of each analysis were considered significant if *p* < 0.05. The data are presented as the mean ± the standard deviation. All statistical analyses were performed using GraphPad Prism software (GraphPad, San Diego, CA).

## Results

The demographic characteristics of the cohort are shown in Table 1. The two groups did not differ with respect to age, sex, or education between baseline and follow-up. Significant differences between HCs and patients with AD were observed for the Mini Mental State Examination (MMSE) scores and Alzheimer’s Disease Assessment Scale-cognitive subscale (ADAS-cog) scores at baseline. Cognitive testing showed a non-significant decrease in the MMSE score, but a significant deterioration in the ADAS-cog score at follow-up compared to at baseline in the AD group. It should be noted that all of the patients with AD were being treated with a cholinesterase inhibitor during the follow-up period.

**Table 1:** Demographic and clinical characteristics of the healthy controls and patients with Alzheimer’s disease. HC = healthy control; AD = Alzheimer’s disease; SD = standard deviation; MMSE = Mini-Mental State Examination; ADAS = Alzheimer’s Disease Assessment Scale-cognitive subscale; ChE = cholinesterase inhibitor.

Characterisic	Healthy control (n = 5)	Alzheimer's disease (n = 5)
**Age at baseline (years), mean±SD**	71.6 ± 4.2	80.4 ± 13.1
**Gender (M/F), n**	4/1	2/3
**Years of education, mean±SD**	15.2 ± 1.8	12.6 ± 1.9
**MMSE at baseline**	28.8 ± 1.8	21.2 ± 2.6
**MMSE at follow-up**	29.0 ± 1.7	20.4 ± 3.4
**ADAS at baseline**	5.1 ± 2.2	19.0 ± 5.1
**ADAS at follow-up**	4.2 ± 1.5	21.4 ± 5.9
**Between PET exams (day)**	519.4 ± 45.0	426.4 ± 1.8
**Treatment at baseline**	none	5 on ChEI

⋆p<0.05 by Mann-Whitney test

**Fig 1:**
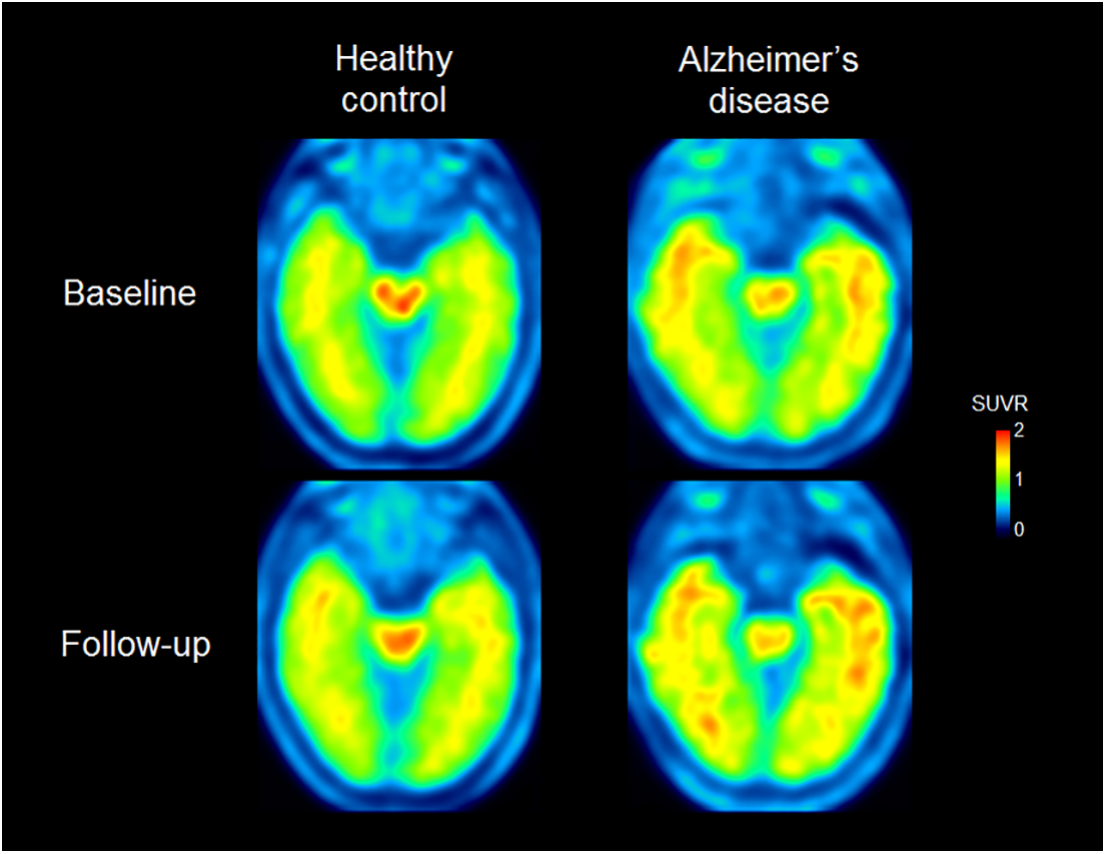
[^18^F]THK-5117 PET images acquired at baseline and follow-up from a HC (left; 78-year-old man, MMSE score of 30 at baseline) and a patient with AD (right; 87-year-old man, MMSE score of 25 at baseline). [^18^F]THK-5117 retention in the anterior and inferior temporal areas is evident in the patient with AD, while it is not in the HC at baseline. In the follow-up images, the [^18^F]THK-5117 distribution is increased toward the posterior temporal region and the retention is higher compared to in the baseline images in the patient with AD. On the other hand, no remarkable change in tracer uptake is observed in the HC images at follow-up compared to in the images at baseline.

**Fig 2:**
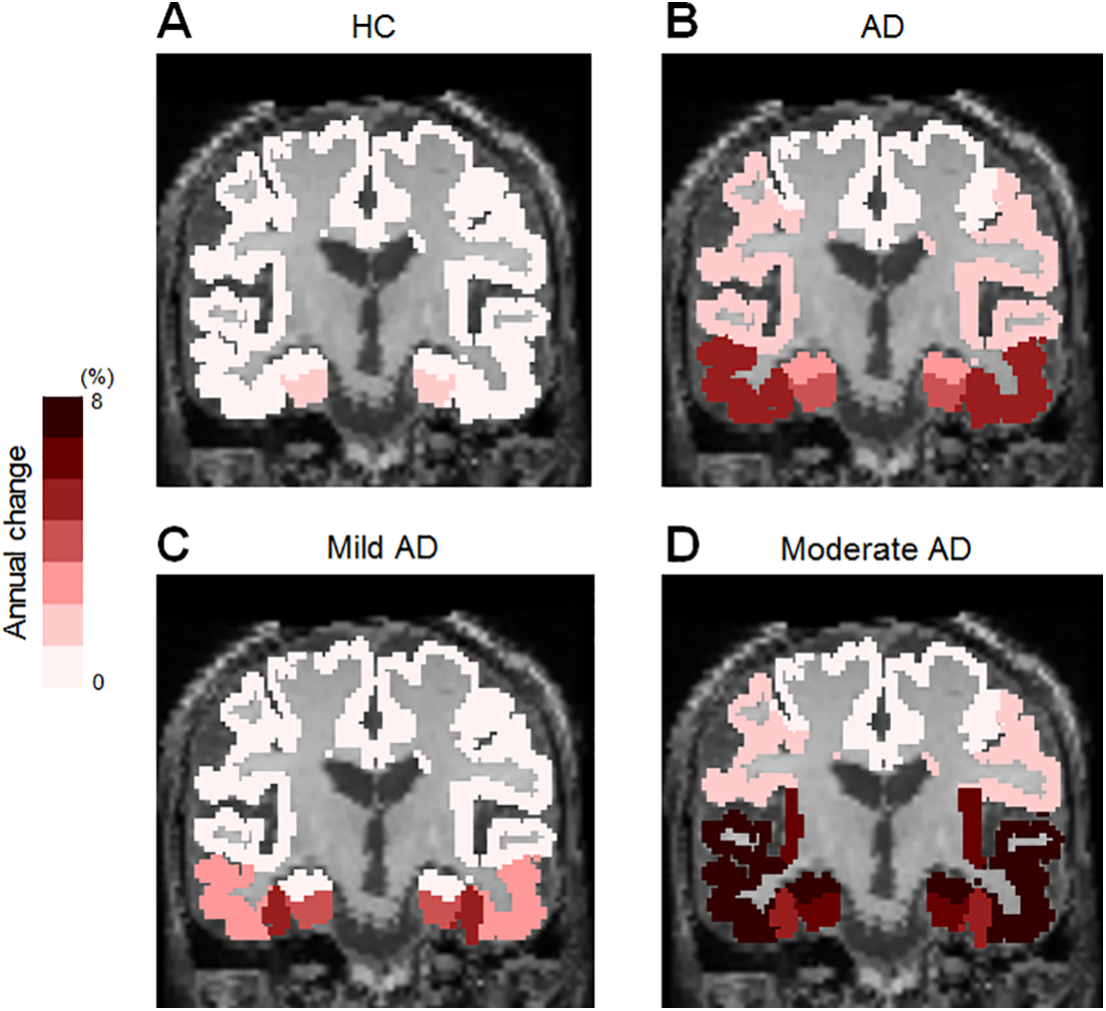
Regional differences in the [^18^F]THK-5117 SUVR annual change in HCs (A) and patients with AD (B). Patients with AD showed greater annual change in the middle and inferior temporal gyri (4.98 ± 3.92%) and in the fusiform gyrus (5.19 ± 2.01%) than did HCs. Compared to the patient with mild AD (57-year-old male, MMSE score of 22 and ADAS-cog score of 12.7 at baseline) (C), the patient with moderate AD (86-year-old female, MMSE score of 18 and ADAS-cog score of 25.3 at baseline) showed greater annual change in the parahippocampal gyrus (7.16%), fusiform gyrus (5.17%), and middle and inferior temporal gyri (8.60%).

Representative [^18^F]THK-5117 PET images at baseline and follow-up are shown in Fig 1. [^18^F]THK-5117 retention in the anterior and inferior temporal regions is evident in the patient with AD (87-year-old man, MMSE score: 25) at baseline, while it is not evident in the HC participant (78-year-old man, MMSE score: 30) at baseline. After one year of follow-up, the patient with AD showed increased tracer uptake in the temporal cortex, while no remarkable changes were observed in the HC participant. The annual change in the [^18^F]THK-5117 retention in the HC and AD groups are shown in Table 2 and Fig 2. The annual percent change in the [^18^F]THK-5117 standardized uptake value ratio (SUVR) in HCs was within 2%. However, patients with AD showed significantly greater annual changes in [^18^F]THK-5117 retention, in the middle and inferior temporal gyri (4.98 ± 3.92%) and in the fusiform gyrus (5.19 ± 2.01%). Patients with moderate AD showed greater SUVR changes in the lateral temporal area than did patients with mild AD (Fig 2).

**Table 2:** Annual rate of change in regional [^18^F]THK-5117 retention ratio for healthy controls and patients with Alzheimer’s disease.

	% annual change of [^18^F]THK-5117 SUVR	
Region, mean±SD	Healthy control	Alzheimer’s disease
Hippocampus	-0.10 ± 1.95	2.55 ± 4.46
Parahippocampal gyrus	1.23 ± 0.82	3.93 ± 3.18
Middle and inferior temporal gyrus	0.44 ± 0.65	4.98 ± 3.92^†^
Fusiform gyrus	0.85 ± 1.75	5.19 ± 2.01^†^
Superior parietal gyrus	-1.77 ± 1.09	0.91 ± 2.97
Lateral occipital gyrus	-1.09 ± 1.11	3.02 ± 1.97

^†^p<0.05 by analysis of variance followed by the Bonferroni’s multiple comparison test.

**Fig 3:**
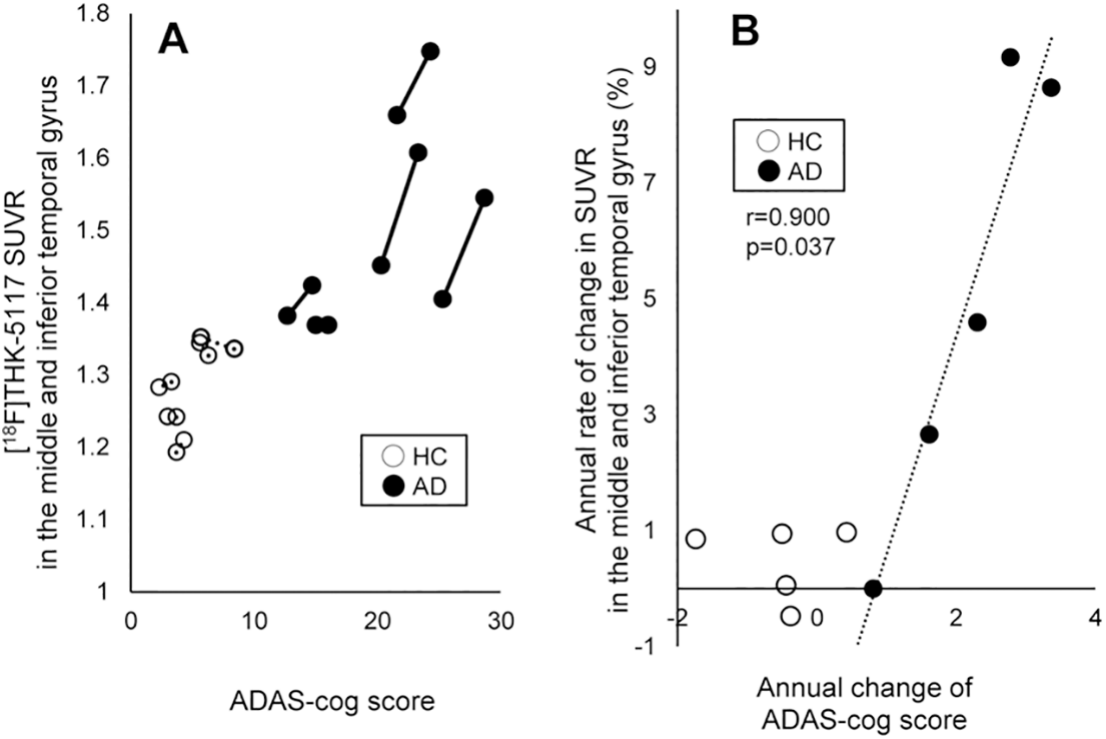
The relationship between the ADAS-cog score and [^18^F]THK-5117 SUVR. (A) Individual changes in the ADAS-cog score and the [^18^F]THK-5117 SUVR in the middle and inferior temporal gyri. Patients with AD (solid line) demonstrate increases in both the ADAS-cog score and [^18^F]THK-5117 SUVR, while HCs (broken line) show no change. Patients with moderate and severe AD show greater [^18^F]THK-5117 retention than do patients with mild AD. (B) Correlation between the annual change in the ADAS-cog score and the annual rate of change in [^18^F]THK-5117 SUVR in the middle and inferior temporal gyri. Filled circles represent the patients with AD and open circles represent the HCs. A significant positive correlation was observed for AD cases (*r* = 0.900; *p* = 0.037).

The relationship between the ADAS-cog score and the [^18^F]THK-5117 SUVR in the middle and inferior temporal gyri is shown in Fig 3A. HCs showed no remarkable changes in their ADAS-cog scores or in the [^18^F]THK-5117 SUVR. In contrast, the three patients with moderate AD showed increased [^18^F]THK-5117 retention compared to HCs and the two patients with mild AD. Correlation analyses were performed to investigate the relationship between the annual changes in cognition and the brain tau load. The results indicated that the annual change in the ADAS-cog score was significantly correlated with the annual change in the [^18^F]THK-5117 SUVR in the middle and inferior temporal gyri (*r* = 0.72; *p* = 0.019) (Fig 3B). Significant correlations were also observed in the fusiform gyrus (*r* = 0.84; *p* = 0.002), parahippocampal and ambient gyri (*r* = 0.67; *p* = 0.033), superior temporal gyrus (*r* = 0.72; *p* = 0.019), posterior temporal lobe (*r* = 0.89; *p* = 0.001), superior parietal gyrus (*r* = 0.64; *p* = 0.048), inferiolateral remainder of the parietal lobe (*r* = 0.86; *p* = 0.048), and lateral remainder of the occipital lobe (*r* = 0.87; *p* = 0.001).

## Discussion

To our knowledge, this is the first report to demonstrate longitudinal changes in the tau pathology in patients with AD. The major findings from this study are as follows: (1) patients with AD showed greater changes in [^18^F]THK-5117 retention in the temporal lobe than did HCs; (2) the spatial pattern of [^18^F]THK-5117 accumulation was consistent with the postmortem observation of tau pathology in the different stages of AD; and (3) the rate of [^18^F]THK-5117 accumulation correlated well with the rate of cognitive decline in patients with AD. These findings suggest that tau PET is a useful technique for investigating the dynamic tau deposition process and its association with cognitive function and neurodegeneration in patients with AD.

In patients with AD, annual changes in [^18^F]THK-5117 accumulation were prominent in the middle and inferior temporal gyri and fusiform gyrus. Post-mortem studies have shown that NFTs initially appear in the transentorhinal area, and then spread to the hippocampus, temporal cortex, and other cortical areas [1,21]. The current PET data suggest that the tau pathology actively spreads from the medial to the lateral temporal cortex during the mild to moderate stages of AD. As shown in Fig 2, the patient with moderate AD showed tau deposition sites that were more widely distributed compared to the patient with mild AD. Considering that the tau pathology is postulated to propagate in a prion-like fashion, the active site of tau deposition may relocate during the course of the disease. In contrast to the increases observed in the fusiform gyrus and lateral temporal cortex, the increase in [^18^F]THK-5117 binding in the hippocampus was modest even in patients with AD (Table 2), suggesting that the tau pathology in the hippocampus reaches a plateau in an earlier stage of AD compared to the pathology in the neocortex. However, it is also possible that brain atrophy in patients with AD may have led to an underestimation of hippocampal [^18^F]THK-5117 binding [22–24]. In future longitudinal analyses of tau PET data, partial volume correction might help to detect small tracer uptake changes in this area.

It has been reported that the annual change in Aβ deposition in the brains of patients with AD is about 3% per year, which is lower than the [^18^F]THK-5117 binding level we observed in the present study. Aβ deposition reaches equilibrium or plateaus in the very early stage of AD [25–27], suggesting that amyloid PET might not be the best tool for identifying the stage of disease severity in patients with AD. Considering the greater annual changes in tau and the stronger association between the tau load and cognition compared to Aβ, tau PET imaging is perhaps more suitable as an indicator of disease progression, even in the advanced stage of AD.

Furthermore, tau pathology progression, as reflected by [^18^F]THK-5117 binding, was significantly associated with cognitive decline in patients with AD (Fig 3b). This result is consistent with previous observations showing that NFTs are highly associated with neuronal loss and with the clinical severity of dementia [17,18], and suggests that the progression of tau pathology is a strong determinant of cognitive decline. To verify the consistency between the [^18^F]THK-5117 distribution, neuronal degeneration, and cognitive decline [22–24], the current tau PET findings should be compared with volumetric magnetic resonance (MR) and 2-[^18^F]fluoro-2-deoxy-D-glucose PET data in the future.

One of the limitations of this study is the small sample size, as only five patients with AD and five HCs were included. Furthermore, the disease stages were restricted to the mild and moderate stages of AD. In the future, longitudinal analysis should be performed with patients in various stages of AD including mild cognitive impairment. In addition, more non-demented elderly people should be examined longitudinally, because tau pathology in the medial temporal cortex is age-related [28–30]. Individuals with subjective cognitive decline are considered to have a high risk of future cognitive decline and conversion to dementia [31,32]. Tau PET has the potential to predict the prognosis of these populations. Longitudinal studies in preclinical patients with AD will further elucidate the potential interaction between Aβ and tau during the course of AD. Therapeutic interventions that utilize disease-modifying drugs would be more effective if these drugs could be administered before the occurrence of irreversible neuronal damage. To facilitate therapeutic trials targeting the Aβ and tau proteins, it is important to detect the underlying pathological process as early as possible [33]. In the present study, [^18^F]THK-5117 was successfully used to visualize the pathologic time course of NFT formation in the human brain. Combinatorial and longitudinal use of amyloid and tau PET imaging has the potential to elucidate the pathophysiology of AD and accelerate the development of disease-modifying drugs in the near future.
